# Missouri Valley Veterinary Association

**Published:** 1896-07

**Authors:** S. L. Hunter

**Affiliations:** Secretary


President.

W. L. RHOADS,

Secretary.

F. S. BRIDGE,

Treasurer.

### MISSOURI VALLEY VETERINARY ASSOCIATION.

THIS Association held its second annual meeting in the parlors of the National Hotel, in Leavenworth, Kansas, Wednesday, June 10, 1896. The meeting was called to order by President Stewart. The following members responded to roll-call: Drs. Bray, Harrison, Hunter, McCurdy, Sihler,

Stewart, Biart, Pritchard, Kaupp, and Payne. Visitors: Drs. T. J. Turner, Day, and Hopkins. The minutes of the February meeting were read and approved. The censors reported favorably upon the applications of Drs. Day, Turner, Hopkins, and Brooking for membership, and they were duly elected.

The Secretary's report was read and ordered filed.

A resolution was presented to change Article I., Chapter 2, of By-laws.

President Stewart then gave his annual address, it being an excellent paper and thoroughly enjoyed by all.

Dr. McCurdy's paper on "Tuberculosis," Dr. Pritchard's views on "Control of Contagious Diseases," Dr. Moore's "Castration of Cryptorchids," Dr. Biart's "Preventive Inoculation for Contagious Pleuro-pneumonia," were all valuable papers, and the discussion upon the same continued until nearly 1 A.M. Great interest was manifested throughout.

The election of officers resulted in the re-election of Dr. S. Stewart, President; Dr. George C. Pritchard, Vice-President; Dr. T. J. Turner, Second Vice-President; Dr. S. L. Hunter, Secretary and Treasurer. Censors, Drs. Sihler, Payne, Hopkins, Bray, and McCurdy.

The following were appointed to prepare papers for our next regular meeting in October: Drs. Hopkins, Day, Harrison, Sihler, G. O. Netherton, J. O. Young, and C. B. McClellan.

Upon motion, duly seconded, the meeting adjourned to meet in Kansas City, Mo., in October.

S. L. Hunter, V.S., D.V.S.,
Secretary.

